# Nanotechnological approach to delivering nutraceuticals as promising drug candidates for the treatment of atherosclerosis

**DOI:** 10.1080/10717544.2021.1892241

**Published:** 2021-03-11

**Authors:** Sindhu C. Pillai, Ankita Borah, Eden Mariam Jacob, D. Sakthi Kumar

**Affiliations:** Bio-Nano Electronics Research Centre, Graduate School of Interdisciplinary New Science, Toyo University, Saitama, Japan

**Keywords:** Atherosclerosis, bio-markers, anti-inflammatory drugs, nutraceuticals, NPs

## Abstract

Atherosclerosis is Caesar’s sword, which poses a huge risk to the present generation. Understanding the atherosclerotic disease cycle would allow ensuring improved diagnosis, better care, and treatment. Unfortunately, a highly effective and safe way of treating atherosclerosis in the medical community remains a continuous challenge. Conventional treatments have shown considerable success, but have some adverse effects on the human body. Natural derived medications or nutraceuticals have gained immense popularity in the treatment of atherosclerosis due to their decreased side effects and toxicity-related issues. In hindsight, the contribution of nutraceuticals in imparting enhanced clinical efficacy against atherosclerosis warrants more experimental evidence. On the other hand, nanotechnology and drug delivery systems (DDS) have revolutionized the way therapeutics are performed and researchers have been constantly exploring the positive effects that DDS brings to the field of therapeutic techniques. It could be as exciting as ever to apply nano-mediated delivery of nutraceuticals as an additional strategy to target the atherosclerotic sites boasting high therapeutic efficiency of the nutraceuticals and fewer side effects.

## 1. Introduction: atherosclerotic development, foam cells, atheroma progression

World Health Organization (WHO) in its study on cardiovascular diseases (CVD) commented that 17.8 million myocardial infarctions (MI) and stroke cases are recorded worldwide every year (World Health Organization, 2015). Earlier it was believed that CVD is an age-related problem affecting only the elderly population, however, Guo et al. conducted elementary research on young adults and found a disturbing fact that a substantial shift has even spread to very young adults aged <30 years (Guo et al., 2018). One of the main reasons behind CVD has been identified as atherosclerosis. Atherosclerosis is a maladaptive coronary disease that is characterized by hardening and narrowing of arteries due to plaque build-up. The arteries are lined by a thin endothelial layer forming an interface between the circulating blood and the inner arterial layer. Under normal conditions, the endothelium keeps the interior of the arteries toned and ensures a smoother blood flow. The narrowing of the coronary arteries limits or slows down the flow of oxygen-rich blood to vital organs leading to heart attacks and strokes collectively called cardiovascular diseases (Tegos et al., 2001).

Reduced shear stress and perturbation in the blood flow at the arteries lead to endothelial cell dysfunction (Deshun & Kassab, 2011). The normal functioning between the smooth muscle cells and the endothelium of arterial vessels is disrupted at the onset of inflammation. Selective solute and cell exchange between the flowing blood and the surrounding tissues is regulated by the vascular endothelium. Small molecules cross the endothelial barrier due to the concentration gradient similarities, whereas the movement of large molecules and cells is permitted only at the impairment of endothelial junctions (Egawa et al., 2013). The pro-atherogenic stimuli and cardiovascular risk factors, such as dyslipidemia, diabetes, obesity, and smoking, cause impairment of endothelial junctions resulting in oxidative stress. Low-density lipoprotein (LDL), one of the most important atherogenic lipoproteins delivers cholesterol (Musunuru & Kathiresan, 2016) to the peripheral tissues (Skålén et al., 2002) which are linked to increased risk of CVD (The Emerging Risk Factors Collaboration, 2009). Endothelial dysfunction allows the entry of LDL into the arterial wall which gets entrapped in the extracellular matrix. Oxidative stress leads to the chemical modification to form oxidative-LDL (Ox-LDL) (Glass & Witztum, 2001), thus activating the endothelium, and up-regulating the vascular cell adhesion molecules (VCAM-1). This is followed by the initial T-cell, monocyte, and leukocyte recruitment (Libby, 2002; Hansson, 2005) which sets the initial stage for atherosclerotic development. Ox-LDL and its oxidized phospholipid components are chemoattractants inducing the expression of monocyte chemotactic protein-1 (MCP-1). MCP-1 attracts monocytes and T-cells that play a fundamental role in the leukocyte recruitment (Falk, 2006) termed as leukocyte extravasation. Monocytes once adhered to the activated endothelial layer, migrate into the intima region differentiating into macrophages with signaling cues received from locally produced monocyte colony-stimulating factor (M-CSF). Macrophage differentiation upregulates scavenger receptor A (SR-A) and CD-36 that mediates the macrophages to assimilate the modified LDL ultimately forming foam cells (Libby, 2002). The yellow discoloration of the arterial wall is the indication of fatty streak formation in atherosclerosis.

**Figure 1.**
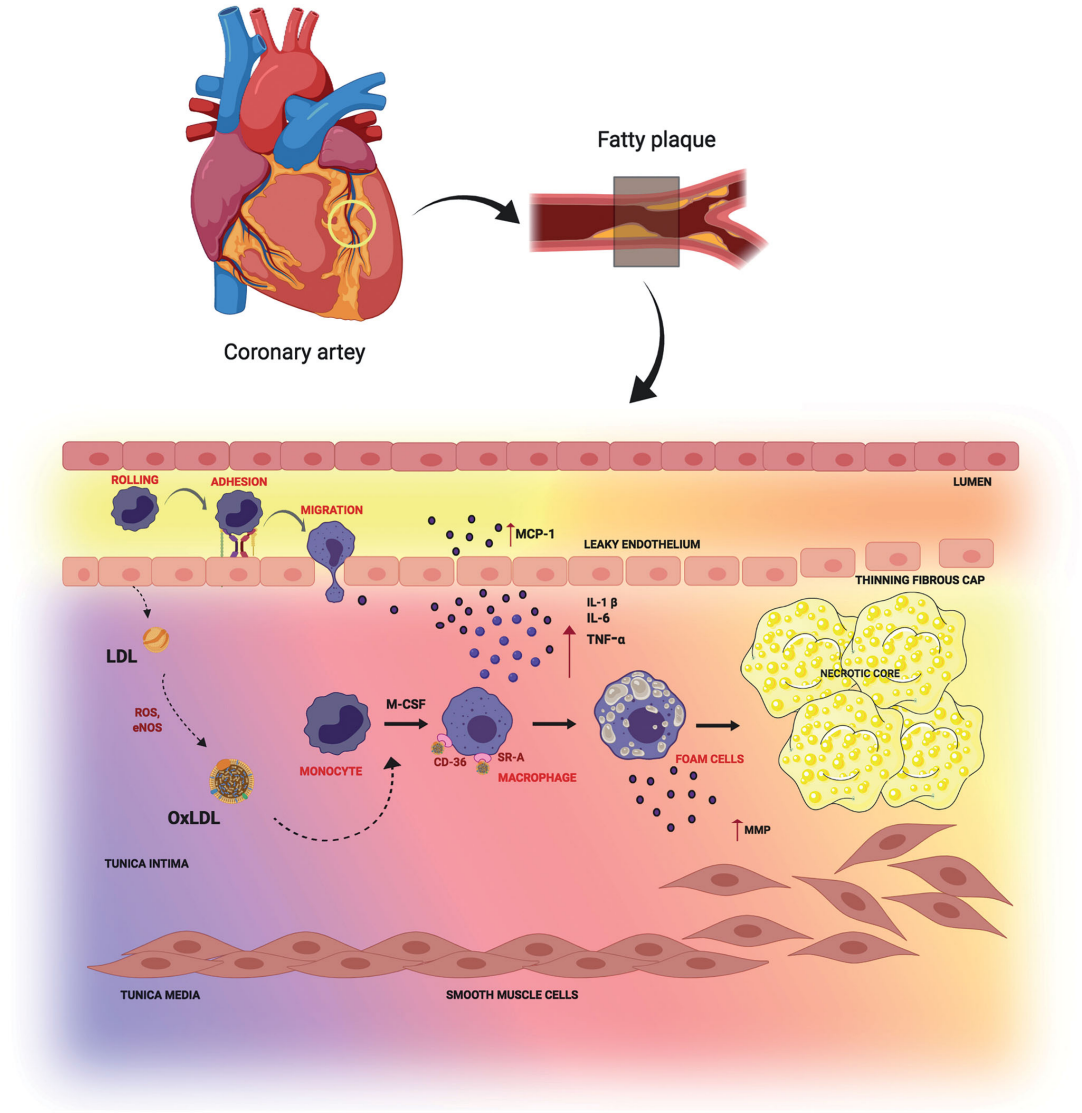
Steps in atherosclerosis initiation and progression: Atherosclerosis is characterized by a yellow color lesion formation. LDL gets converted to Ox-LDL after reaction with reactive oxygen species (ROS). Monocytes travel through leaky endothelium and differentiates into macrophages. Macrophage assimilates the Ox-LDL to form foam cells. Calcification and monocyte recruitment cause smooth cell proliferation and forms a necrotic core.

**Figure 2.**
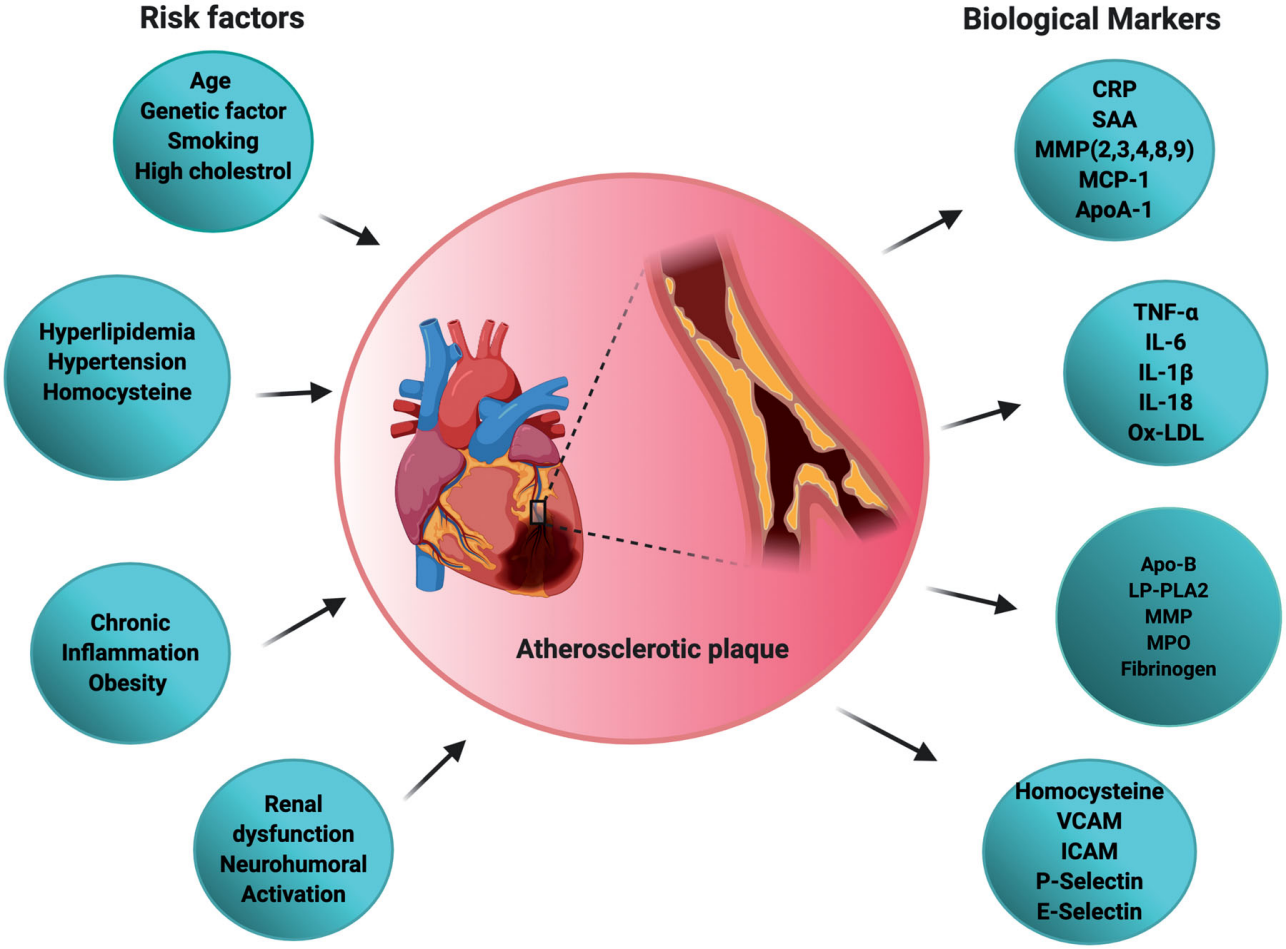
Schematic representation of different risk factors and biomarkers associated with atherosclerosis useful in disease diagnosis and progression.

Atheroma progression is mediated by smooth muscle cell migration and proliferation along with the activation of nuclear factor-kappa B cells (NF-κ B), and release of inflammatory cytokines interleukin-1β (IL-1β), tumor necrosis factor- α (TNF-α), IL-6, as well as CC-chemokine ligand 5 (CCL5), CXC motif-chemokine ligand 1 (CXCL1) and CXCL3. As the fibrosis continues, the oxygen supply to the macrophages gets reduced and eventually undergo apoptosis. These apoptotic cells gradually result in the formation of an acellular fibrous capsule with a lipid-laden core and drive coronary thrombosis. Coronary thrombosis encompasses plaque disruption and plaque erosion (Moreno, 2001). Increased lipid content, infiltrated macrophages, and a thin fibrous cap constitute disrupted plaques whereas eroded plaques comprise an intact fibrous cap laden with smooth muscle cells. Plaque disruption occurs when the cap is thinned/fractured by infiltrated macrophage-derived foam cells (Peter, 1995). Interstitial collagen molecules confer most of the tensile strength to the protective fibrous cap by resisting proteolytic degradation. The intact collagen fibrils undergo proteolytic cleavage by interstitial collagenases like matrix metalloproteinases (MMP)-1, MMP-8, and MMP-13 leading to plaque disruption and thrombosis in the atheromatous plaques (Sukhova et al., 1999; Newby, 2007) (Figure 1). A complex cascade of events that comprises several risk factors, activation of biomarkers, inflammation, matrix remodeling, and myocyte injury contributes to the development and progression of atherosclerosis depicted in Figure 2. The currently available drugs are aimed at treating atherosclerosis functions by inhibiting the inflammatory pathways and cholesterol metabolism. There is a growing body of evidence that has proven the efficacious nature of the present therapies as a first-line treatment against atherosclerosis. But as the age-old saying goes ‘a boon and a bane’ conventional therapies possess their share of bane, which have hindered them from achieving their ultimate therapeutic potential. Some of the major issues that afflict the present atherosclerotic treatments include liver and kidney damage, diabetes, off-target effects, low bioavailability, drug–drug interactions, and inability to effectively reach the target sites. Hence a rational approach would be to design better drugs to circumvent such issues, resort to appropriate delivery vehicles, and also apply complementary therapies. A significant amount of literature focuses on the different types of nutraceuticals proven to exert immense benefits in combating atherosclerosis prevention and progression (Moss & Ramji, 2016; Ruiz-León et al., 2019). However, not much has been said about their clinical translation and therapeutic efficacy against the disease. Though nutraceuticals delivery via nano-drug delivery systems (NDDS) has shown exceptional results in the field of anti-cancer therapy and neurodegenerative diseases (Masoudi Asil et al., 2020; Salama et al., 2020) their application in CVD is yet to be explored. Delivery of these nutraceuticals to the atherosclerotic lesion-prone site will be a challenging task owing to inadequate absorption in the gut, rapid degradation, and clearance from the body. Henceforth, the idea behind this review is to provide an outlook toward the translation of these remarkable natural compounds using nanotechnology, and the advantages of currently established nano-systems being used in atherosclerosis via passive and active targeting for improved treatment.

The advent of nanotechnology has demonstrated success in the treatment of atherosclerosis in recent years to an extent by providing better delivery vehicles for the currently available drugs to carry out their targeted action and minimize off-targets. Also, nanocarriers are exploited for imaging purposes to monitor inflammation and disease progression. With the emerging research to explore newer targets in atherosclerosis, such as molecular and cell-specific receptors, these could be further employed in the development of innovative atherosclerotic nanomedicines. The majority of the nanomedicines that have been experimentally studied are based on endothelial cell targeting, macrophage targeting, lipid content, angiogenesis, and thrombosis. These strategies often utilize the conventional drugs in the nanocarrier to achieve the therapeutic purpose albeit it is never too late to have complementary treatment options such as nanoparticle-mediated delivery of nutraceuticals ensuring improved disease outcome (Flores et al., 2019).

## 2. Established therapeutic modalities

To identify the vulnerable plaques, several noninvasive imaging technologies such as ultrasound (US), computed tomography (CT), magnetic resonance imaging (MRI), and nuclear imaging (i.e. single-photon emission computed tomography (SPECT) and positron emission tomography (PET) were developed and in current use. The asymptomatic nature of vulnerable plaques limits the usage of the current imaging modalities to detect the instability. Quitting smoking (reduced ROS), avoiding saturated and trans-fat from the diet (lowered LDL), regular exercise (related to increased laminar flow, and reduced LDL) could be the stepping stone to prevent atherosclerosis. All said, atherosclerosis being a lifetime condition with limited cures, the best option could be to slow down the disease progression or halt the condition from worsening. In this section, we discuss recent therapeutic modalities to treat atherosclerosis and also some of the related drawbacks associated.

### 2.1. Surgical methods

Percutaneous coronary intervention (PCI), also known as coronary angioplasty, is performed by opening narrowed coronary arteries to place a stent thus improving the blood flow to the heart and mitigating chest pain. Coronary artery bypass grafting (CABG) involves the grafting of a new artery to bypass the narrowed coronary arteries while boosting blood flow and preventing heart attacks. In the list of surgical methods, carotid endarterectomy is a common surgical process that involves the correction of the internal carotid artery by removing plaque build-up eventually restoring the blood flow to the brain. Surgical procedures of the blood vessel-blockade have achieved clinical success for many years, yet are also associated with numerous complications such as restenosis, in-stent restenosis, and late-stage clotting to name a few (Giannini et al., 2018).

### 2.2. Anti-inflammatory medication

Anti-inflammatory drugs, for example, cholesterol-lowering drugs (statins, fibrates, and bile acid sequestrants, etc.), angiotensin-converting enzyme (ACE) inhibitors, beta-blockers, calcium channel blockers, and anti-thrombosis drugs like Aspirin are considered to be first-line therapies for various cardiovascular disease conditions. The statins class of drugs when designed were regarded as the perfect marriage between the drug and atherosclerosis that inhibit 3-hydroxy-3-methylglutaryl-coenzyme A (HMG-CoA) reductase enzyme in the cholesterol pathway finally lowering the cholesterol levels in addition to providing anti-inflammatory actions (Rosenson, 2004; Ehrenstein et al., 2005). However, continued statin treatment is usually suggested even after the target cholesterol level is reached for sustained protection against atherosclerosis. The boon of statin therapy often comes with its share of limitations of causing hepatotoxicity, renal toxicity, the onset of type-2 diabetes mellitus, and other conditions (Bitzur et al., 2013). Fibrates and niacin treatment supplemented with statin therapy reduce high and small dense LDL particles, and triglyceride (TG) levels while beneficial effects of lipoprotein subtraction profile are also observed in hypertriglyceridemia patients. Despite demonstrating enhanced efficacy of both the drugs in the lowering of TG levels, there are still innumerable reports concerning the fact that no significant reductions in cardiovascular events have been achieved (Ito, 2015). Cholesterol conversion into bile acids is an attractive strategy to lower cholesterol levels and ameliorate atherosclerosis. Bile acid sequestrants (BAS) like cholestyramine and colestipol represent the class of drugs approved for hypercholesterolemia that binds to bile acid in the lumen of intestines to form non-absorbable complexes facilitating the excretion of fecal bile acid. This will lead to the subsequent conversion of cholesterol into bile acids in the liver (Insull, 2006).

The process of atherogenesis involving the renin-angiotensin system though has been hypothesized through various experimental findings yet to be well-defined to elucidate further molecular mechanisms. Angiotensin production from endothelial cells is regulated by angiotensin-converting enzyme (ACE) which in turn exerts control over events like smooth muscle growth and proliferation during atherosclerosis also causing vascular wall injury (Ambrosioni et al., 1992). Therapeutic blockade of ACE will prevent the formation of angiotensin II leading to lowering of blood pressure levels while improving heart conditions. ACE inhibitors assist in the stabilization of unstable plaque in atherosclerotic lesions by modulating local endocrine and inflammatory pathways (Higgins 2003). ACE inhibitors have been classified into three main groups based on their chemical structure: (1) sulfhydryl-containing ACE inhibitor (Captopril), (2) dicarboxylic-containing ACE inhibitors (Benzapril, Enalapril, Quinapril to name a few), (3) phosphorus-containing ACE inhibitor (Fosinopril). ACE inhibitors except for Enalapril in general are administered intravenously in 1.25 mg dosage every 6 h with an optimal increase to 5 mg (Herman et al., 2019). The mode of action of these inhibitors is not yet fully known however they lower the production of angiotensin II from angiotensin I that reduces blood pressure, prevents smooth muscle and cardiac myocytes remodeling, and promotes natriuresis. ACE inhibitors also come with their fair share of adverse effects by causing angioedema in the intestines, tongue, glottis, and larynx obstruction leading to life-threatening conditions (Korzeniowska et al., 2017). Sustained elevated arterial blood pressure aggravates and accelerates atherosclerosis as confirmed by mounting evidence in the past few decades (Bronte-Stewart & Heptinstall, 1954).

Anti-hypertensive drugs though have not yet presented any well-known effects during the course of hypertension diuretics but are among the widely used drugs to treat hypertension as monotherapy or in combination with other drugs to provide additive effects in reducing blood pressure and counteract the water and salt retention in the body. A class of anti-hypertensive drugs is the beta-blockers such as propranolol that acts by blocking the cardiac beta-adrenergic receptors to decrease the cardiac output in addition to antianginal actions (Bühler et al., 1972).

Anti-thrombotic therapy is a sought-after treatment option for thrombosis that underlies acute coronary syndrome. The appropriate management of reduction in platelet aggregation is warranted for anti-thrombotic drugs. Aspirin is one of the keystone anti-thrombotic drugs that has been demonstrated to reduce fatal and non-fatal risk of myocardial infarction in at least 50% of patients. Aspirin interferes with the formation of thromboxane A2 and reduces platelet aggregation via the blockade of the cyclo-oxygenase pathway. A beneficial dose of 75–50 mg seems to have a sustained effect with lower side effects related to gastrointestinal conditions (Watson et al., 2002). Clopidogrel and Ticlopidine belong to the adenosine diphosphate (ADP) inhibitors class of anti-thrombotic drugs (Sharis et al., 1998). Ticlopidine has previously been shown to reduce infarction, stroke, and angina for at least six months nonetheless associated with reversible neutropenia and thrombocytopenia. Clopidogrel represents the advanced derivative version of ticlopidine which is highly effective in reducing platelet aggregation. Clopidogrel also reports decreased risk of bleeding than aspirin and has better tolerability. Anti-thrombotic therapy additionally comprises of other strategies such as glycoprotein IIb/IIIa receptor inhibitors, anti-coagulant therapy (heparin treatment and fibrinolytic treatment) that has elucidated varied results in patients of the acute coronary syndrome in different experimental settings (Watson et al., 2002).

## 3. Alternative approaches: nutraceutical derivatives

A famous concept formulated by the French epidemiologists known as the ‘French Paradox’ observes the low incidence of coronary heart disease irrespective of a diet comprising of high dietary cholesterol and saturated fats. An explanation for the French paradox could be due to the potential moderate intake of wine (Ferrières, 2004). The term nutraceutical is an amalgamation of the terms ‘nutrition’ and ‘pharmaceutic’ that comments on the discipline related to the health benefits of food and food products including prevention and treatment against diseases (Biesalski, 2002). Several clinical and pre-clinical reports describe the preventive nature of nutraceuticals on the onset and progression of atherosclerosis, however, to shed further light into the mode of action outlining their importance in the prevention of atherosclerosis studies related to their dose and mode of administration, pharmacokinetics, and pharmacodynamics should be carried out. In this section, we discuss a few of the nutraceuticals that have been implemented as a preventive strategy in the progression of atherosclerosis through extensive experimental findings and also shown in Table 1.

**Table 1.** List of nutraceuticals experimentally studied for CVD including atherosclerosis.

Nutraceuticals	Benefits	Clinical observations in humans
Curcumin	Decreased the proinflammatory cytokines such as IL-1β, IL-6, and tumor necrosis factor in human monocytes (Abe et al., 1999) Promotes the polarization of anti-inflammatory M2 macrophage phenotype in murine macrophages (Gao et al., 2015) Reduced oxidative stress and LDL oxidation(Ramírez-Tortosa et al., 1999) Reduced atherosclerotic lesion size by 50% in Apo-E and LDL double-knockout mice after 4 months (Olszanecki et al., 2005)	Reduced arterial stiffness (Chuengsamarn et al., 2014) Improved endothelial function (Akazawa et al., 2012)
Hydroxytyrosol	Reduced the expression of the proinflammatory adhesion proteins ICAM1 and VCAM1 in HUVECs (Dell’Agli et al., 2006) Hydroxytyrosol oil-based diets lower LDL level and increase plasma HDL level in Wistar rats (Mangas-Cruz et al., 2001) Reduced plaque size in hyper-lipidemic rabbits (González-Santiago et al., 2006)	Reduced serum Ox-LDL levels (Fitó et al., 2008) Reduced inflammatory biomarkers and improved endothelial function (Widmer et al., 2013)
Omega-3 PUFAs	Reduced the expression of pro-atherogenic markers in both murine and human macrophages (Hughes et al., 1996) Increased the expression of cholesterol efflux genes (ABCA1, ApoA1) and decreased the expression of LDL-uptake genes (SR-A1, CD36) (Song et al., 2013)	Increased plaque stability and reduced levels of proinflammatory cytokines (Niki et al., 2016) Increased endothelial function and reduced arterial stiffness (Tousoulis et al., 2014) Reduced atherothrombotic risk (Franzese et al., 2015)
Flavanols	Reduction in endothelial exocytosis, which activates endothelial cells to produce pro inflammatory cytokines (Yamakuchi et al., 2008) Reduce atherosclerotic lesion size by regulating the genes involved in cell migration (Morrison et al., 2014)	Increased vasodilatation, reduced Ox-LDL levels (Tinahones et al., 2008) Reduced expression of proinflammatory cytokines (Hsu et al., 2007) Enhanced endothelial function and reduced arterial stiffness (Heiss et al., 2015)
Omega-6 PUFAs	Reduce the mRNA level of ICAM-1,VCAM-1 and reduce the size of atherosclerotic plaque size (Takai et al., 2009) DGLA(dihomo-gamma linoleic acid) metabolizes to prostaglandin E_1_ via COX pathway imparting anti-atherosclerotic effect (Takai et al., 2009).	Reduced serum LDL, total cholesterol, and triacylglycerol levels (Guivernau et al., 1994)
Resveratrol	Reduced foam cell formation by inhibiting Ox-LDL uptake and increasing cholesterol efflux in human THP-1 macrophages (Voloshyna et al., 2013). Reduced atherosclerotic lesion size by approximately 50% (Berbée et al., 2013)	Reduced risk of cardiovascular event (Levantesi et al., 2013)
Vitamin C	Improved endothelial function by enhancing nitric oxide synthase activity (Matsumoto et al., 2003)	Reduced risk of a cardiovascular event (Khaw et al., 2001) Increase endothelial function (Ashor et al., 2014)
Allicin	Attenuates the expression of proinflammatory cytokines including IL-1β, IL-6, and tumor necrosis factor in murine macrophages treated with lipopolysaccharide (Lee et al., 2012) Reduce the leukocyte adherence to the endothelium (Zanardo et al., 2006) Reduce the production of ROS in *in vitro* with supplementation of H_2_S and attenuates the expression of CD36, ACAT1 and MSR1 (Zhao et al., 2011)	Clinical trial: Attenuated plaque volume (Koscielny, 1999) Reduced the progression of coronary calcification (Budoff et al., 2004)
Berberine	Reduced the proinflammatory gene expression, including MCP-1, IL-1β, IL-6, iNOS (Jeong et al., 2009) Decreased the migration of macrophage cell into the lesion areas (Cheng et al., 2015) Upregulate the expression of cholesterol efflux gene (ABCA1) (Lee et al., 2010) Reduced the expression of VCAM-1, ICAM-1(Wang et al., 2011)	Reduced serum total cholesterol, LDL-cholesterol, and HDL-cholesterol levels (Kong et al., 2004)
Carnosine	Reduced glycation of LDL, resulting in reducing intracellular cholesterol accumulation in human macrophages (Rashid et al., 2007). Improved plaque stability in Apo-E deficient murine with diabetes (Brown et al., 2014) Increase plaque stability by increasing collagen content (Brown et al., 2014)	Preserved insulin sensitivity and secretion with no change in blood pressure and other CVD risk factors (de Courten et al., 2016)
Coenzyme Q10	Attenuated LDL oxidation, resulting reduce foam cell formation by increasing cholesterol efflux via miR-378 (Wang et al., 2014)	Improved endothelial function (Gao et al., 2012)
Short chain fatty acids (SCFA)	Reduce the formation of nitric oxide and proinflammatory cytokine IL-1β, IL-6 and tumor necrotic factor (Liu et al., 2012) Decrease leukocyte adhesion by inhibiting the production of VCAM-1 (Menzel et al., 2004) Reduced macrophage migratory capacity and ameliorate plaque stability (Aguilar et al., 2014)	Reduced risk of a CHD (Pereira et al., 2004)

Nutraceuticals are functional foods or dietary supplements possessing health benefits that could be administered to individuals that face the borderline risk of developing CVD. Polyunsaturated fatty acids (PUFA) like omega-3 PUFAs and omega-6 PUFAs attenuates atherosclerotic plaque development and progression by reducing the pro-atherogenic markers experimentally reported earlier in murine and human macrophages studies (Hughes et al., 1996; Miles et al., 2000). Omega-3 PUFAs food sources that include fish oils and flax seeds have been clinically relevant in improving cardiovascular health in patients with marginal hyperlipidemia by reducing the blood triacylglycerol according to a meta-analysis that was published in 2015 (Leslie et al., 2015). The health benefits of consuming omega-3 PUFAs enriched foods show impressive results through epidemiological pieces of evidence along with conflicting conclusions. For extensive reading on the superiority of PUFAs in controlling atherosclerotic plaque, the reader is directed to the review by Ramji D (Ramji, 2019). Polyphenols are a group of compounds that has the structural feature of phenolic hydroxyl groups and are further differentiated into various classes based on their primary aromatic rings, oxidation status, and presence of certain functional groups in their structure. These compounds are traditionally divided into four groups namely flavonoids, lignans, phenolic acids, and stilbenes that are profoundly present in most of the plant and plant products including vegetables, fruits and nuts, herbs, tea, and wine (García-Villalba et al., 2010; Tangney & Rasmussen, 2013). Polyphenols exert their beneficial effects via the regulation of numerous biochemical and signaling pathways working independently or in conjunction to achieve desired efficacy. In terms of providing athero-protection, polyphenols have been demonstrated to operate through different postulated mechanisms comprising of decrease in LDL oxidation, improvement in endothelial function, increased release of the potent vasodilator nitric oxide (NO) modulation of lipid metabolism, and impart antioxidative properties by scavenging free oxygen and nitrogen among the few (Tangney & Rasmussen, 2013). Flavonoids among the group of polyphenols have been researched quite well due to their anti-inflammatory actions against CVD. Flavonoids bear a three-ring structure which is sub-divided into the classes of flavanols, flavanones, flavan-3-ols, flavones, anthocyanins, and isoflavones (Marzocchella et al., 2011). Specific flavonoids such as quercetin inhibit the production of lipid hydroperoxide (LOOH) that in turn regulate the LDL oxidation by macrophages thus initiating a stepping stone in the blockade of foam cell formation in the atherosclerotic process (Kostyuk et al., 2011). Polyphenols have also been shown to modify the release of vasodilators NO and endothelium-derived hyperpolarizing factor (EDHF) from the endothelium consequently relaxing the vessels demonstrated extensively from a plethora of experimental studies (Schini-Kerth et al., 2010). Cohort studies in the past have indicated a decline in the age-related coronary heart disease (CHD) mortality rates upon the increased intake of flavonoid-rich foods (Peterson et al., 2012).

Resveratrol, a polyphenolic compound from the class stilbene is primarily a constituent of red wine. Resveratrol owing to its anti-inflammatory and anti-oxidative properties is shown to have cardioprotective benefits by protecting LDL against peroxynitrite oxidation (Borriello et al., 2010). Several epidemiological studies have shown that a Mediterranean diet comprising of large quantities of olive oil and nuts daily has displayed beneficial effects and lowered the incidence rates of atherosclerosis progression (Kromhout et al., 1989; Keys, 1997; Sofi et al., 2014). Olive oil contains numerous polyphenolic compounds including oleuropein, tyrosol, hydroxy-tyrosol, and some lignans (Granados-Principal et al., 2010). The anti-inflammatory effects of olive oil are mostly exerted by the compound oleuropein that has exemplified the reduction in ROS-mediated MMPs and cyclooxygenase-2 (COX-2) expression in HUVEC cells (Scoditti et al., 2012). Oleuropein in conjunction with hydroxy-tyrosol has also managed to dysregulate the lipopolysaccharide (LPS) mediated ICAM-1, VCAM-1, and E-selectin expression in HUVEC cells (Carluccio et al., 2003). Unfortunately, when the production of olive oil is done using fully ripened olives, the oleuropein undergoes degradation thus imparting limited health benefits. The concentration of hydroxy-tyrosol increases throughout the ripening process projecting it to be the most potent anti-atherogenic compound present in olive oil (Soler-Rivas et al., 2000). The PREDIMED study conducted in Spain 2013–2019 involving 7447 participants potentially having a higher CVD risk were put on a 5-year Mediterranean diet with supplementation of olive oil and nuts, that positively delineated a decrease in the rates of cardiovascular events thus elucidating the cardioprotective health benefits of olive oil consumption (Estruch et al., 2013). Additional studies on the anti-atherogenic status of consuming phenol-enriched olive oil have elaborated its importance in reducing total cholesterol and plasma-LDL (Gorinstein et al., 2002) levels and increased HDL levels in Wistar rats when compared to non-enriched virgin olive oil diet (Mangas-Cruz et al., 2001).

A popular nutraceutical supplement called the red yeast rice (RYR) produced by fermenting rice (Oryza sativa) with the fungus *Monascus purpureus* is known to control cholesterol levels. The fermented rice due to its distinctive red color has a group of molecules called monacolin K (lactone) and monacolin Ka as open ring acid forms that undergo interconversion in the body (Younes et al., 2018). Monacolin K shares chemical similarities with the commercial statin lovastatin and henceforth possesses the ability to inhibit HMG-CoA reductase. Administering monacolin K at a dose of 3–10 mg/day reduced 20–25% LDL concentrations with negligible effects on HDL levels (Li et al., 2014; Fogacci et al., 2019). Even though monacolin K is readily bioavailable than lovastatin and belongs to the safe and natural spectrum of nutraceuticals its adverse effects such as hepatic and gastrointestinal distress cannot be neglected. Moreover, statins and RYR co-administration should be avoided to manage pharmacodynamic effects (Cicero et al., 2017).

Phytosterols/phytostanols (PS) are the group of triterpenes commonly found in vegetables and derivatives like oils, nuts, grain derivatives, and other non-vegetable sources such as egg yolks and crustaceans (Katan et al., 2003). Among the 250 molecules of PS available, β-sitosterol is the most common, and abundantly found in the plant products. PS, in general, are not synthesized by humans, and hence keeping the concentrations of phytosterols to an optimum level through proper diet would be a requirement to achieve the therapeutic benefits. PS reduces the cholesterol levels by competing with it that leads to lower cholesterol solubilization into micelles to be absorbed by the intestinal tract. The low absorption of cholesterol facilitated its fecal excretion provided a continuous supplementation of phytosterols is warranted to achieve the required cholesterol-lowering effects (Ras et al., 2014). The anti-inflammatory effects of PS have been observed in a pre-clinical study of Apo-deficient mice fed on a high-fat diet with 2% phytosterol supplementation for 2-weeks. The mice fed with the phytosterol-enriched diet exhibited a 60% decrease in atherosclerotic lesion size followed by a reduction in the pro-inflammatory cytokines (IL-6 and TNF-α) production and elevated levels of the anti-inflammatory cytokine IL-10 (Nashed et al., 2005). A recommended serving of five or more fruits and vegetables proves to manifest the beneficial health effects of the PS properties.

Garlic consumption as a health supplement has proven to demonstrate anti-atherogenic and anti-inflammatory effects in clinical studies. Garlic contains a natural organic sulfur compound called allicin which is converted from the parent compound alliin by the enzyme alliinase. Allicin due to its unstable nature, breakdown into smaller polysulfides to form hydrogen sulfide (H_2_S) which then attributes anti-inflammatory action (Wang, 2012). H_2_S has been shown in murine models to mitigate the leukocyte adherence to endothelium thus culminating in the process to reduce the inflammatory response (Zanardo et al., 2006). Preliminary clinical studies have revealed upon administration of aged garlic extract atherosclerotic development is decelerated in comparison to statin therapy whereas (Budoff et al., 2004) in a meta-analysis of 45 trials reported a drop in the LDL serum levels, triacylglycerol, and cholesterol levels after 1–3 months of garlic supplementation (Ackermann et al., 2001). An important component of Iranian traditional medicine is the inclusion of Barberry that has the active component Berberine known to possess anti-bacterial, anti-fungal, anti-inflammatory, improved gastric stimulation, reduced hypersensitivity, and improved digestive tract (Dong et al., 2013). Due to its poorly bioavailable nature berberine could be incorporated into microemulsions and nanoparticles (NPs) to tackle such issues and fully exploit its therapeutic properties (Li et al., 2009; Khemani et al., 2012). The effect of berberine to lower plasma cholesterol is a potential sign to deliver anti-atherosclerotic action depending on genetics and levels of basal cholesterolemia as reported in an earlier review (Johnston et al., 2017). Berberine also demonstrated to have a prebiotic effect to mediate a microbiota-induced anti-atherosclerotic action in animal studies (Zhu et al., 2018).

**Figure 3.**
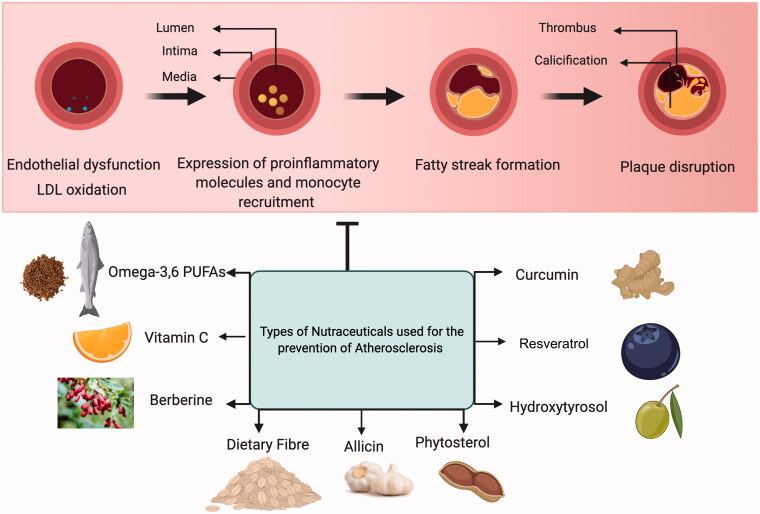
Schematic illustration of various kinds of nutraceuticals experimentally studied to prevent/treat atherosclerotic disease development and progression.

Flavanols, for instance, green tea catechins and cocoa flavanols have shown promising results as nutraceuticals in an attempt to prevent atherosclerotic development by attenuating the expression of several pro-inflammatory cytokines like IL-6 receptor, IL-8, TNF-receptor 2, and endothelial exocytosis (Hsu et al., 2007; Yamakuchi et al., 2008). Dietary supplementation of vitamin cocktails (vitamin-C and vitamin-E) has been shown to decrease the prevalence of coronary artery disease (CAD) and influence prospective cardiovascular health benefits (Osganian et al., 2003; Gavrila et al., 2005). Adding to the list of beneficial nutraceuticals, curcumin (Olszanecki et al., 2005), carotenoids such as astaxanthin (Kishimoto et al., 2016) and lycopene (Arab & Steck, 2000), and use of probiotics (Cavalcanti Neto et al., 2018) have immensely contributed in their distinctive manner in regulating the progression of atherosclerosis by employing diverse biological properties including anti-oxidative, decreased LDL and pro-inflammatory levels, etc. Figure 3 shows a schematic representation of the different types of nutraceuticals used in atherosclerosis treatment.

## 4. Nanomedicines and drug delivery in atherosclerosis diagnosis and treatment

**Figure 4.**
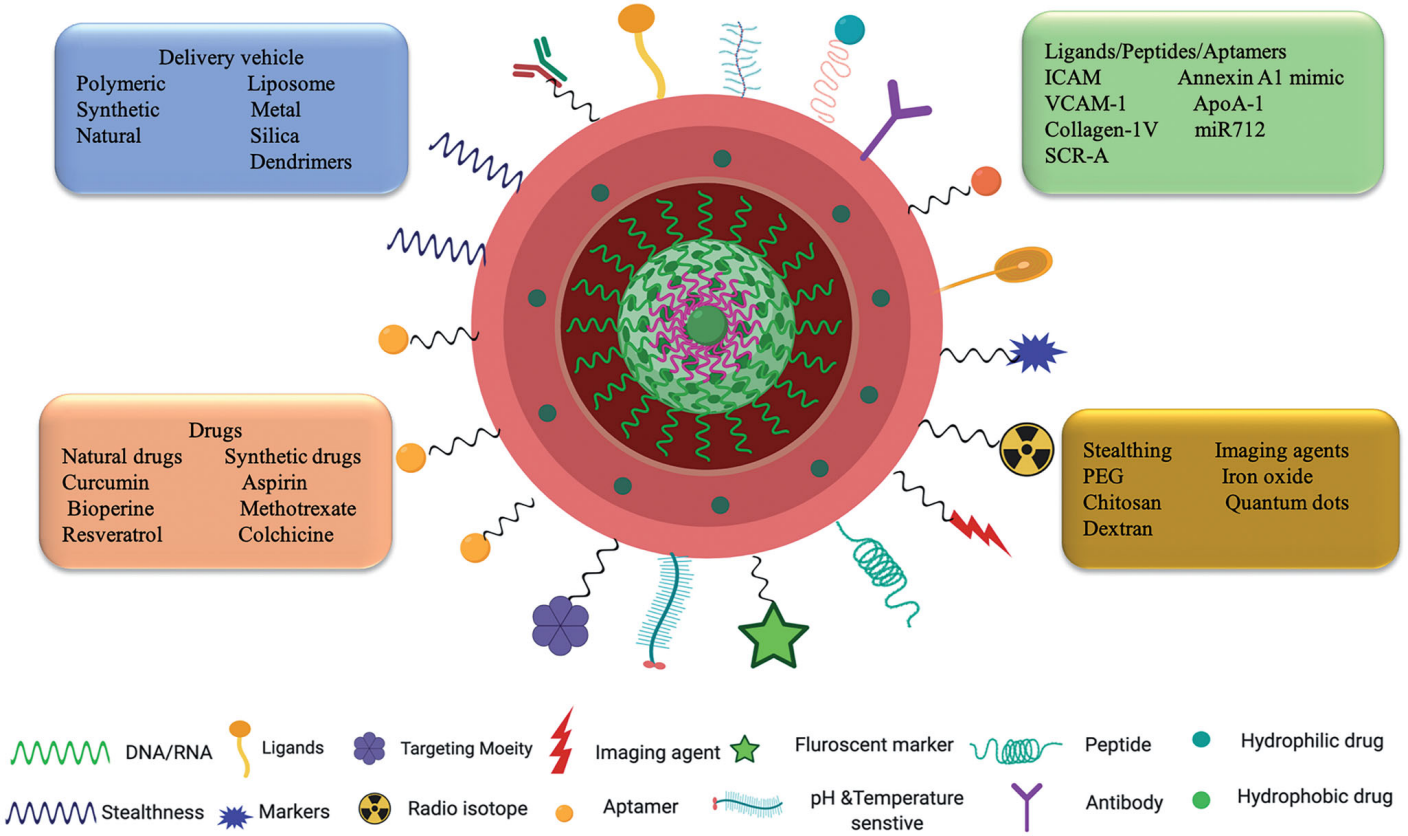
Nanotechnology and targeted drug delivery: Schematic representation of a nano-drug delivery system encapsulated with active molecules and surface modified with different targeting moieties having both therapeutic and diagnostic abilities.

The field of nanotechnology is on a growth spree and is contributing to every walk of life. Precise control in the fabrication of NPs and their physicochemical properties diversifies the clinical application of these agents in the medical field. (Lobatto et al., 2011). Nanomaterials owing to the size difference with their macroscopic materials alters their physical, chemical, optical, and mechanical properties. Due to the nanometer size range, NPs have the added advantage of several biological implications such as evasion of the immune system, enhanced cellular internalization, and ability to interact with cells and tissues. NDDS ensures safe delivery of drug products such as chemical and bioactive molecules improving pharmacokinetics and ensuring limited side toxicities. An ideal NDDS should be fabricated in such a way that it reaches its target site without any hindrance from the *in vivo* physiological environment to carry out its intended function and be able to administer locally as well as systemically. Adjustments made to the NDDS to improve their bioavailability, biocompatibility, drug loading efficacy, and surface modifications will cater to the specific biomedical applications (Qiu & Bae, 2006). NPs provide an environment for the incorporation of hydrophilic and hydrophobic agents in a polymeric core or into the lipid layer respectively. To improve cell-specific targeting, prevent immunogenicity and blood circulation, the surface of the NPs can be modified with peptides, polymers or antibodies, biodegradable hydrophilic polymers like PEG, chitosan, and dextran to name a few (Schiener et al., 2014). An example of a nanocarrier loaded with chemical and bioactive molecules surface modified with different targeting moieties is depicted in Figure 4.

NPs had their early success story as drug delivery carriers in cancer therapy applications for encapsulating anti-cancer compounds having hydrophobic nature for better biodistribution, specificity, and to reduce side effects (Bertrand et al., 2014). As mentioned earlier nutraceuticals delivered via NPs is one of the success stories for anti-cancer therapies. The commonly known nutraceuticals that have been packaged into nano-formulations to fight against cancer are curcumin, piperine, resveratrol, green tea catechins, and polyphenols, etc. (Nair et al., 2010). Nano-formulations of these compounds contributed to their enhanced bioavailability and superior therapeutic efficacy (Nair et al., 2010). Curcumin NPs are one of the nutraceutical-based nano-formulations that have achieved great accomplishments as a cancer therapy and chemo-preventive measure in a wide range of cancers experimentally (Yallapu et al., 2013). The limitations of polyphenols including the short half-life and low bioavailability were solved by the development of nano-encapsulated polyphenols, which were then investigated for their anti-cancer therapeutic efficacy (Squillaro et al., 2018). A recent study from our lab has demonstrated the superior efficacy of BioPerine, a derivative from black pepper, when encapsulated inside a PLA-chitosan/PEG, coated nanoparticle displayed enhanced cytotoxicity against multi-drug-resistant breast cancer and significantly downregulated P-glycoprotein (P-gp) expression in the cancer cells. The BioPerine NPs reportedly showed better efficacy than the commercial P-gp inhibitor Verapamil in the treatment against multi-drug-resistant breast cancer cells (Pillai et al., 2020). Neurodegenerative disorders (NDs) are burdened with the lack of effective treatment regimens, complicated disease etiology that includes genetic factors, environmental factors, and increased oxidative stress. Since polyphenols are blessed with anti-oxidative properties, their delivery via NPs such as nanospheres, nano-capsules, solid-lipid NPs, liposomes, etc. would exert neuroprotective actions more effectively by modulating various pro-inflammatory genes. These nano-systems can cross the blood–brain barrier (BBB) effectively to release the payload at the targeted site of action (Squillaro et al., 2018). Curcumin-loaded nanoliposomes demonstrated the effective inhibition on the *in vitro* formation of fibrillar amyloid-β_1–42_ (A β_1–42_) peptide compared to free curcumin and their derivatives (Taylor et al., 2011).

**Figure 5.**
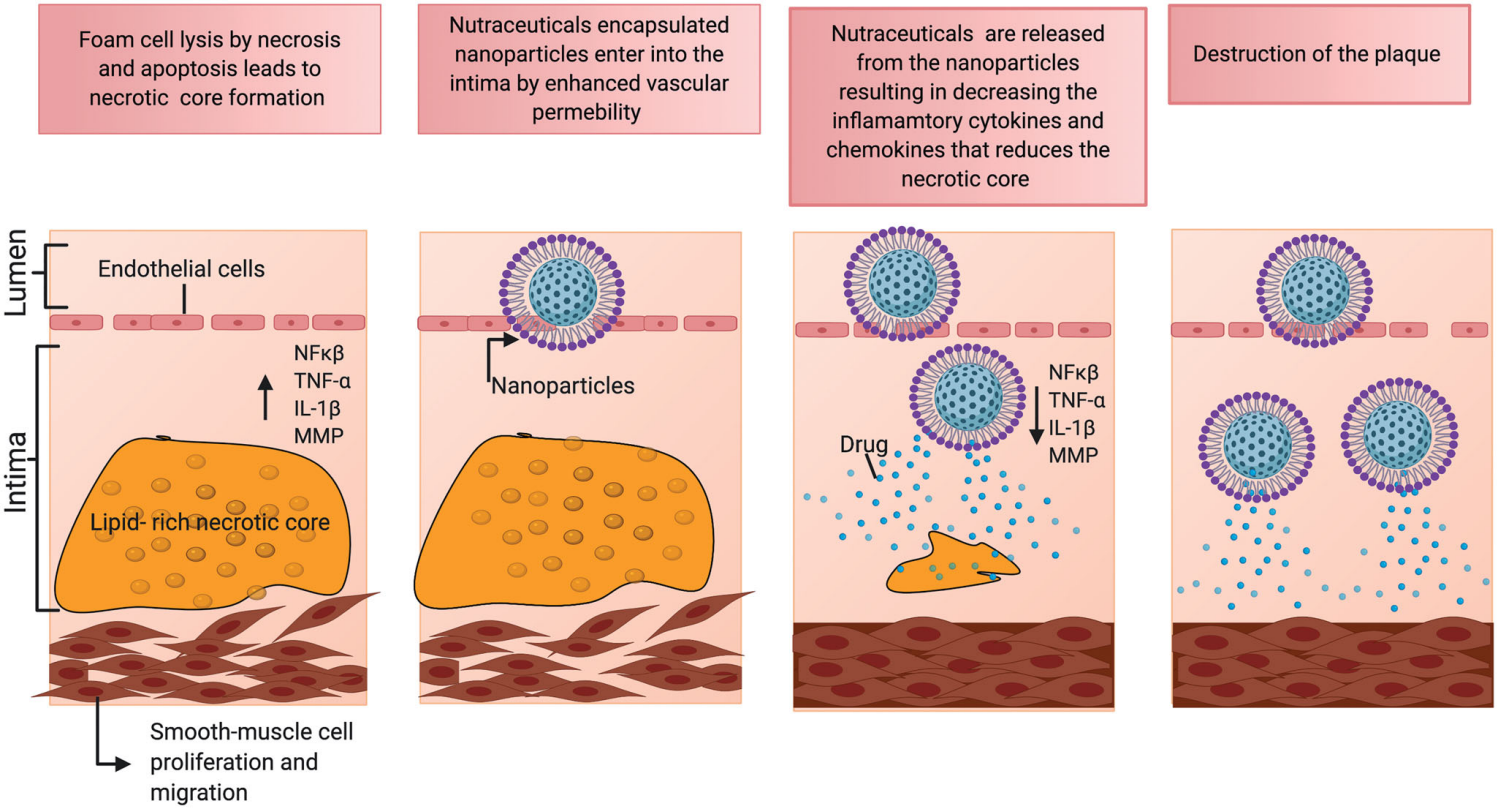
Schematic representation of nano-nutraceuticals when administrated to atherosclerosis disease site will improve their therapeutic efficacy due to the enhancement in bioavailability.

Insightful knowledge from the previous oncological and NDs-based applications extrapolated toward the management of cardiovascular diseases via nanomedicine strategies. NPs are suited perfectly to combat atherosclerosis as drug delivery carriers in the various phases of the disease. Nanocarriers developed for clinical applications like imaging and drug delivery against atherosclerosis comprise both organic and inorganic nanomaterials or a combination of both. Previous reviews on nanotechnological applications in atherosclerosis have majorly stressed the delivery of conventional therapeutics using nanoparticulate systems, targeting the biomolecules on the plaque sites, and imaging techniques. In their review, Jayagopal A et al. discussed the progress made in the late 2000s on the clinical management of atherosclerotic plaque using nanomaterials dedicated to therapy and diagnosis (Jayagopal et al., 2010). A recent comprehensive review divulged the progress of nanotechnology made so far in vascular diseases, including atherosclerosis, and its associated complications (Flores et al., 2019). This review summarized the different nanomedicine-based vascular therapies designed to target macrophages, prevent inflammation, inhibit plaque neovascularization, altering lipid metabolism, and targeting thrombosis demonstrated preclinically in *in vivo* models. Each of these nano-systems constructed was carriers of conventional drugs, anti-siRNA, inhibitors, and diagnostic agents, which were either investigated as therapeutic agents or theranostic agents against atherosclerosis. The authors also talked about the challenges faced in the development and how the nanomedicines could be potentially translated into clinics (Flores et al., 2019). Another area in the realm of nanotechnology-based atherosclerotic applications is to monitor the disease progression using nanomaterials. Atherosclerosis is a slowly progressing disease where the gradual build-up of plaque leads to severe underlying consequences. Zhang Y et al. discussed the possibilities of exploiting the full potential of nanomaterials as theranostic agents for atherosclerosis management by providing a personalized, efficient, and image-guided treatment plan for the patients (Zhang et al., 2019). In our review, we have tried to encompass the additional treatment strategies utilizing nutraceuticals and how their nano-mediated delivery strategies will add to the growing list of new generation and innovative nano-therapies for atherosclerosis. Some of the commonly used NPs for atherosclerosis as a measure for both diagnostic and therapy includes iron-oxide NPs, superparamagnetic (SPIONs), small superparamagnetic iron oxide (USPIO), gold NPs, polymer NPs, and lipid NPs respectively (Bejarano et al., 2018) and these can be essentially used as carriers for nutraceutical delivery. Figure 5 shows the schematic representation of a potential nano-nutraceutical that could be administered in the treatment of atherosclerosis.

### 4.1. Iron oxide NPs and gold NPs

The application of nanomaterials in the diagnosis of atherosclerosis using techniques of magnetic resonance imaging (MRI), computed tomography (CT), biomarker detection, and photoacoustic imaging was possible due to the diverse range of properties they possess. The first usage of NPs for cardiovascular diseases began in the early 90 s with the iron-oxide NPs as MRI vascular contrasting agents (Weissleder et al., 1990). These NPs include both SPIONs and USPIO that have been revealed to provide positive insights for diagnosing atherosclerotic plaque due to their outstanding longer blood half-life and MRI contrast agents (Weissleder et al., 1990). SPION AMI-25 was compared with another dextran-coated USPIO (2–5 nm) where the USPIO demonstrated enhanced capillary wall permeability and also lower uptake by the mononuclear phagocyte system (MPS). Improvements in their bioavailability and tissue interaction were done by coating with a variety of polymers and biomolecules also known as stealth NPs that prevent the attraction of blood proteins toward the nanoparticle surface (Weissleder et al., 1991). SPION AMI-25 (Endorem; Laboratoire Guerbet, Aulnaysous-Bois, France) a stable colloidal aqueous magnetic (Fe_3_O_4_) suspension coated with low-molecular dextran was the first magnetic NPs for vessels imaging using MRI. SPION AMI-25 were biodegradable NPs having a size range from 120 to 180 nm (Wang, 2011). Following these investigations on the advantages of applying USPIO and SPIONs as MRI contrast imaging agents, several modifications have been added to the NPs and used for atherosclerosis associated inflammatory changes (Ruehm et al., 2001), detection of macrophages in human plaques (Kooi et al., 2003), and CD163 macrophage marker detection in atherosclerotic intraplaque lesions (Tarin et al., 2015). Matuszak et al. attempted to target the arterial vessels *in vivo* utilizing lauric acid/albumin-coated SPIONs (Matuszak et al., 2018). The group demonstrated that surface-modified SPIONs under the influence of external magnetic field gradient were readily accumulated in the *ex vivo* and *in vivo* rabbit atherosclerotic model by additionally possessing superior biocompatibility and colloidal stability. The group pursued further under the impression that utilizing the respective SPIONs as a drug delivery carrier to deliver certain immunomodulatory agents like Dexamethasone would alleviate the inflammatory response in the plaque formation stages. The SPIONs conjugated with Dexamethasone (SPION-DEXA) on contrary to the authors’ hypothesis increased the burden of the inflammatory response in the developed plaques (Matuszak et al., 2018). The authors finally concluded that SPIONs as a magnetic drug delivery carrier could be tested using drug candidates like statins to counteract the paradoxical results of SPION-DEXA. Several investigations related to the usage of SPIONs as MRI contrasting agents have long been established followed by current research pushing the envelope far enough in developing more superior SPION-based MRI probes visualization of atherosclerotic plaque. Evans RJ et al. developed a range of SPIONs with different core sizes and coated with poly (maleic anhydride-alt-1-octadecene) (PMAO) and poly (ethyleneimine) (PEI) or alendronate for application as MRI probes. Among the different MRI contrast agents developed the lead SPION agent coated with PMAO had a core size of 10 nm and was shown to rapidly accumulate in the vascular regions of elastin enriched plaque (Evans et al., 2020). The surface coating provided a negative charge to the nanoparticle that maximized its circulation time and limits its liver uptake. Noninvasive techniques for the detection of vulnerable plaques and their stability in the atherosclerosis (AS) process will provide an edge over the early diagnosis of CVD. Superparamagnetic iron oxide NPs decorated with anti-IL-6 (Anti-IL-6 USPIO) when administered to an AS rabbit model with a damaged abdominal aortic intima manifests superior targeting of IL-6 and detecting vulnerable plaque both *in vitro* and *in vivo* conditions. This study provides an innovative noninvasive approach to evaluate CVD risks (Mo et al., 2020).

**Figure 6.**
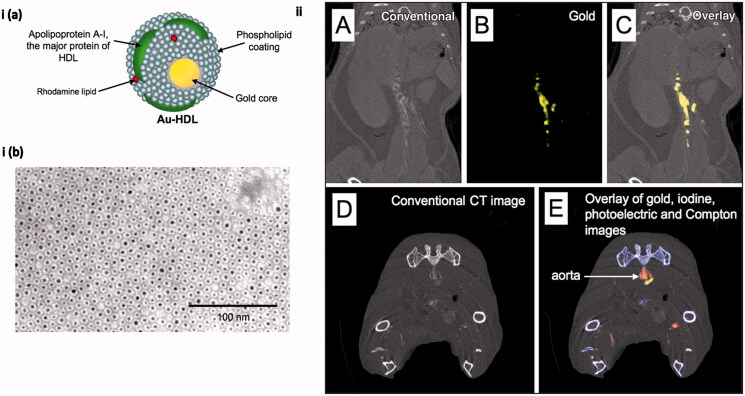
(i(a)). Schematic illustration of macrophage-targeted gold core nanoparticle Au-HDL. HDL = high-density lipoprotein. (i(b)) Characterization of Au-HDL on negative-stain transmission electron microscopy (TEM) image. (ii(A–C)), Spectral CT images of thorax and abdomen in apo E–KO mouse injected 24 h earlier with Au-HDL. (ii(D, E)), Spectral CT images near bifurcation of aorta in apo E–KO mouse injected with Au-HDL and an iodinated emulsion contrast agent (Fenestra VC) for vascular imaging. (Reproduced with the permission from D. P. Cormode et al. [Cormode et al., 2010]. Copyright 2010 Radiology).

Coronary artery stenosis grading and determining plaque calcification in atherosclerosis need robust monitoring and accurate diagnostic techniques such as CT with additional improvements made using NPs with unique properties. The presence of macrophages in atherosclerotic plaque that leads to plaque destabilization and thrombus formation posits macrophages as an interesting choice for early detection by CT. The earliest attempt in this direction to monitor macrophages in atherosclerotic plaque using CT was made by Hyafil et al. who developed iodinated polymer NPs. The use of iodinated polymer NPs showed immense potential in detecting the atherosclerotic plaque from surrounding tissue after the injection of the NPs (Hyafil et al., 2007). The drawbacks associated with the iodinated polymer materials such as renal clearance and toxicity, vascular permeation were solved with the development of PEG-coated gold NPs by Kim, Park, et al. (2007). These NPs provided a much clearer depiction of cardiac ventricles and vessels. Another improvement made with the gold NPs was the detection and imaging of macrophages in atherosclerotic plaque and vasculature respectively using gold-high density lipoprotein (HDL) NPs shown in Figure 6 (Cormode et al., 2010). Gold NPs also offer the possibility of conducting photoacoustic imaging due to the presence of free charge oscillation on its surface mediated by the exciting wavelength that produces enhanced optical absorption and contrast (Li & Chen, 2015). Several studies were conducted to unravel the immense potential of gold NPs with varied modifications as a source for the detection of early inflammation in endothelial cells and targeting atherosclerotic plaque by photoacoustic technique (Kim, Huang, et al., 2007; Wang et al., 2009).

### 4.2. Polymeric NPs and lipid NPs

Advancements in the nanoparticulate-based drug delivery system have contributed significantly to atherosclerotic therapeutics. Drug delivery systems (DDS) could be potentially harnessed to target atherosclerosis due to their sustained drug release properties, easy surface modifications with antibodies, peptides, and aptamers, and also size, shape, and surface chemistry. PLGA based drug delivery carriers feature as one of the sought-after nanosystems for atherosclerotic treatment owing to their controlled drug release (Kamaly, Yameen, et al., 2016) and biocompatible properties. FITC-loaded PLGA NPs were used for coating the surface of stainless-steel stents which function as drug delivery carriers. This nanoparticle-eluting-stent system showed superior delivery of the FITC marker into the stented porcine coronary artery reported by Nakano et al. (Nakano et al., 2009). PLGA nanosystems were also used to carry drug payload to atherosclerotic plaque lesions as investigated in two independent studies. Pitavastatin-loaded PLGA NPs inhibited plaque destabilization and rupture in Apo E**^−/−^** mice (Katsuki et al., 2014), and pioglitazone-loaded PLGA NPs were designed as a means to the homing of inflammatory Ly-6C^high^ monocytes to cause the suppression of proteinase activity and IL-6, MMP-9, and EMMPRIN expression that ultimately leads to plaque rupture (Nakashiro et al., 2016). Active targeting ligands such as monoclonal antibody against elastin attached to the PLA NPs facilitates specific targeting strategy toward the degraded elastic lamina as shown successfully in three different vascular disease models of rodents (Sinha et al., 2014). Polyphenolic compounds pose as attractive targets for atherosclerosis treatment imparting anti-inflammatory and anti-oxidative properties. Ferulic acid is a potent antioxidant that has been used to synthesize NPs with anti-oxidant polymeric cores decorated with amphiphilic macromolecules (PFAG) targeting scavenger receptors to analyze its therapeutic efficacy against athero-protection by minimizing Ox-LDL uptake and decelerate foam cell formation (Chmielowski et al., 2017). The controlled release of ferulic acid from the PFAG NPs generated excellent athero-protective nature by inhibiting Ox-LDL uptake amongst the other ferulic acid intermediate polymeric nano-formulations. The PFAG NPs downregulated the gene expression of macrophage scavenger receptors such as MSR-1, CD-36, and LOX-1 and minimized ROS levels in human monocyte-derived macrophages (HMDM) (Chmielowski et al., 2017). Chronic inflammation is an underlying disease pathology in atherosclerosis that could be bridled with the delivery of anti-inflammatory cytokine IL-10. Targeted polymeric NPs encapsulating IL-10 (Col-IV IL-10 NP22) was the potent nanoparticle that displayed a significant reduction in acute inflammation, prevented plaque formation, and decreased the formation of necrotic cores (Kamaly, Fredman, et al., 2016). The endothelial barrier integrity in atherosclerosis is compromised leading to subsequent lesion and plaque formation because of immune cells and macromolecules infiltration (Beldman et al., 2019). Hyaluronan NPs (HA-NPs) were investigated to trace their fate in the plaque microenvironment. Using super-resolution and correlative light and electron microscopy, it was revealed that 75% of enriched HA-NPs were localized in the endothelial junctions and the endothelium. This study shed light on nanoparticle trafficking across atherosclerotic endothelium and its modulation (Beldman et al., 2019). Combating oxidative stress by redox-based therapies could obstruct the progression of atherosclerosis. Maiocchi S et al. reported the translation of redox-based therapies by using two different NPs to address redox dysregulation and inflammation in atherosclerosis. One of the NPs incorporates a redox-active drug molecule Nrf2-activator CDDO-Methyl inside polymeric NPs (CDDO-Me-NPs), and the other encapsulates an anti-inflammatory compound oleic acid (OA) inside LDL-like NPs (LDL-OA-NPs) (Sophie Maiocchi et al., 2020). The NPs were developed with the aim of passive targeting against atherosclerotic plaque, which demonstrated to selectively reduce the plaque formation in the murine Apo E**^−/−^** and LDLr**^−/−^** models, thus confirming the enhancement of redox-based interventions and anti-inflammatory activity in atherosclerosis (Sophie Maiocchi et al., 2020). Dendrimer-based polymer NPs synthesized using methoxy poly (ethylene glycol)-b-poly(e-caprolactone) copolymer encapsulating Curcumin (Cur) is a step in the translation of nutraceutical-based nano delivery approaches which effectively showed a reduction in the atherosclerotic lesion and stabilized the vulnerable plaques in Apo E**^−/−^** mice (Meng et al., 2019).

Apart from the proven advantages of polymeric drug delivery carriers, lipid-based nanoformulations are not far behind from posing as eminent candidates for atherosclerotic plaque targeting. Amidst its capacity as a biocompatible material to encapsulate both hydrophobic and hydrophilic drug payload, lipid NPs also have a superiority for atherosclerotic treatment because of their cholesterol sequestration ability from circulating lipoproteins (Rodrigueza et al., 1998). Lobatto et al. demonstrated that the delivery of synthetic glucocorticoid prednisolone via PEGylated liposomal NPs to a rabbit model of atherosclerosis efficiently reduced inflammation locally compared to the free-treated liposomal NPs. The group further emphasized that prednisolone half-life increased to 45–63 h when delivered through liposomal NPs than the free intra-venous (IV) administration of prednisolone (Lobatto et al., 2010). Liposomal NPs encapsulating small interfering RNA (siRNA) against the chemokine receptor CCR2 led to the reduced accumulation of monocytes/macrophages in atherosclerotic plaque areas (Leuschner et al., 2011). Apolipoprotein (Apo B) is a protein constituent of lipoprotein and which is essential for the transport and metabolism of cholesterol. The elevated Apo B and LDL correlate with the risk of CAD. Since conventional therapies are limited in their efficacy to target Apo B, the delivery of liposome encapsulating anti-Apo B siRNA can silence Apo B to reduce the circulatory LDL (Zimmermann et al., 2006). Proprotein convertase subtilisin/kexin type 9 (PCSK9) regulates the expression of LDL receptors. Overexpression of human PCSK9 increases the circulating LDL cholesterol, whereas deletion of PCSK9 has the reverse effect. The therapeutic silencing of PCSK9 by delivering liposome NPs encapsulated with anti-PCSK9 siRNA reduced the plasma LDL cholesterol concentration up to 60% within 48 h (Frank-Kamenetsky et al., 2008). Berberine an isoquinoline quaternary alkaloid showed to have cardioprotective benefits (Zhang et al., 2014) were formulated into liposomal NPs to evaluate the IL-6 secretion inhibition after MI in C57BL/6J mice (Allijn et al., 2017). Liposomal berberine formulation was highlighted to preserve substantial cardiac ejection fraction 28 days post-MI compared to free berberine hence laying a foundation for the application of berberine nanoformulation to treat atherosclerosis.

Besides polymer and lipid NPs, another drug delivery carrier that attracts attention due to its intrinsic affinity toward atherosclerotic plaque is the high-density lipoprotein (HDL) platform. The HDL nanocarrier offers the flexibility to load drugs in the hydrophobic core and the phospholipid layer (Damiano et al., 2013). HDL NPs were exploited to carry cholesterol-lowering oral drugs such as statins (simvastatin) in a study conducted by Duivenvoorden et al. (Duivenvoorden et al., 2014). Anti-inflammatory agents like statins exacerbate side-effects in patients if given beyond a certain dosage. Hence the delivery of simvastatin via HDL NPs in an Apo E**^−/−^** mouse model of atherosclerosis showed improved accumulation in the lesion while preventing plaque progression with a low dose for a 3-month treatment period (Duivenvoorden et al., 2014). In addition to the existing HDL NPs, hybrid PLGA/HDL NPs were developed as second-generation NPs by microfluidics technology to aid better-controlled drug release. These hybrid NPs, when administered to knockout Apo E**^−/−^** mouse model, indicated increased accumulation in the atherosclerotic plaques (Sanchez-Gaytan et al., 2015). Other innovative NPs to combat atherosclerosis include the development of platelet-like NPs (PLNs) and cationic lipoparticles. PLNs possess platelet-like characteristics such as discoidal morphology and adhesion are reported to migrate toward the vascular wall and vascular injury sites to reduce the bleeding time in wound injuries (Anselmo et al., 2014). Cationic lipoparticles incorporating anti-miR 712 in its core and a peptide targeting VCAM-1 decorating its neutral coat were delivered to the inflamed aortic mouse endothelial cells in *in vitro* and *in vivo* conditions to report the prevention in atheroma formation in atherosclerosis (Kheirolomoom et al., 2015).

### 4.3. Biomimetic NPs

Another class of NPs that have attracted considerable interest in the development of next-generation nano-based therapies for atherosclerosis are cell-derived biomimetic NPs that are shown to have effective immune evasion features, longer blood circulation time, and specifically targets the atherosclerotic sites (Fang et al., 2014; Yang et al., 2020). Along with their high biocompatible nature, biomimetic NPs also successfully transfer the phospholipid bilayers from natural cells to NPs keeping the surface proteins intact (Narain et al., 2017). In terms of application in CVD, biomimetic NPs are divided into three types: (1) whole cells, (2) cell-membrane, and (3) vesicles-based NPs (Jin et al., 2018). Atherosclerosis is a disease of the circulatory system channelizing the advantages of whole cells such as RBCs, platelets (Wu et al., 2016), neutrophils (Han et al., 2018), and monocytes (Lee, 2014) to deliver therapeutic compounds of choice is a potential outlook toward developing alternative drug-delivery vehicles. These whole-cell delivery methods possess the precedence over traditional NPs in terms of prolonged circulation time (Zaitsev et al., 2006; Burnouf et al., 2013) secretion of cytokines (Woollard & Geissmann, 2010), larger loading capacity (Xue et al., 2019) easy to scale up preparation, and administration followed by enhanced accumulation at the target site. Extracellular vesicles (EV) from platelets and neutrophils have shown in several cardiovascular diseases to have superior targeting ability at inflammatory sites inferring EV-mediated delivery of therapeutic compounds to atherosclerotic sites holds promise (Shah et al., 2018). Zhang et al. in 2011 developed the cell membrane coating technology that coats the traditional NPs with natural cell membranes providing an advantage to the NPs to exploit the cell’s surface antigen diversity and escape immune capture (Hu et al., 2011). A notable contribution in the area of using biomimetic NPs against atherosclerosis treatment was recently explored by Gao C et al. Macrophage membrane was utilized to decorate the surface of ROS-responsive NPs fabricated using amphiphilic oxidation-sensitive chitosan oligosaccharide (Oxi-COS). The biomimetic NPs were formed via the self-assembly process and a model drug atorvastatin was incorporated into the NPs. The macrophage membrane will assure the evasion of the NPs from the reticuloendothelial system (RES) and suppress inflammation by the sequestration of pro-inflammatory cytokines. The ROS-responsiveness of the NPs will lead to specific drug release at the targeted site and improve therapeutic efficacy (Gao et al., 2020). Biomimetic NPs along with inorganic and organic NPs have been currently studied in a wide array of different disease scenarios including CVD holds an intriguing premise to deliver nutraceuticals against atherosclerotic treatments. Table 2 lists the examples of nanomedicines that have been experimentally studied in atherosclerosis.

**Table 2.** List of nanomedicine-based approaches studied in CVD including atherosclerosis.

NPs Used	Target	Result	Reference
Hydrophobic mucic acid core and polystyrene amphiphilic core shells M_12_PEG and PS_15_PEG	Scavenger receptor-A (SR-A) and Scavenger receptor B (CD36)	Directly compete with the uptake of oxidized LDL via scavenger receptors SR-A and CD36. NPs uptake led to a downregulate in the gene expression of both receptors.	(Petersen et al., 2014)
Polymeric Micelle encapsulated LXR agonist (GW3965)	Scavenger receptor A	The nanoscale macromolecules decreased intimal cholesterol levels (macromolecule alone 50%; macromolecule encapsulated GW3965 70%) and decrease the presence of macrophage near the site of injury	(Iverson et al., 2011)
Lipid nanoparticle-encapsulated CCR2-siRNA	Monocytes/macrophages	NPs were co-localized in monocytes lowered the migratory capacity of monocytes toward MCP-1. The silencing of CCR2 reduces the myocardial infarct size after coronary artery occlusion and reduced the number of monocytes/macrophages by 82%.	(Leuschner et al., 2011)
Phosphatidylserine (PS)-presenting liposomes containing iron oxide	Phosphatidylserine receptor (PSR)	PS-presenting liposomes enhanced the expression of mannose receptor CD206, increased secretion of anti-inflammatory cytokines TGF-β and IL-10 and downregulated proinflammatory markers CD86.	(Harel-Adar et al., 2011)
Single-walled carbon nanotubes (SWNT) modified by Cy5.5	Monocyte cells	SWNT-Cy5.5 were co-localized with atherosclerotic macrophages. Light (808 nm) exposure of ligated carotid arteries induced apoptosis in macrophages, but not in control arteries without light exposure or SWNTs.	(Kosuge et al., 2012)
Dextran Nanoparticle (DNP) loaded with C-C chemokine receptor type 2 – small interfering RNA (CCR 2 siRNA)	Macrophage cells	Significant reduction in the PET signal in the aortic root and arch of Apo E**^−/−^** mice treated with siCCR2. Decreased PET signal led to a reduction in inflammatory gene expression including CCR2	(Majmudar et al., 2013)
HDL mimic NPs containing the core of PLGA, cholesteryl oleate, and a phospholipid bilayer decorated with triphenylphosphonium (TPP) cations (TPP-HDL-ApoA-I-QD NPs)	Macrophages	NPs improved reverse cholesterol transport as shown in *in vitro* studies. TPP-HDL-Apo A-I-QD NPs significantly reduces the total cholesterol and triglyceride compare to control.	(Marrache & Dhar, 2013)
PLGA-PEG-collagen IV encapsulating with Ac2-26. (annexin A1mimetic peptide act on G protein coupled receptor)	AnnexinA1(N-formyl peptide receptor FPR2/ALX)	Ac2-26 NPs are more potent than Ac2-26 native peptide limiting the recruitment of neutrophils (56 % vs 30 %). Ac2-26 NPs significantly blocks the tissue damage in hind-limb ischemia-reperfusion injury by 30 % compared with controls.	(Kamaly et al., 2013)
Lipid emulsion Di dodecyl-Methotrexate (LDE-ddMTX)	LDL receptor (low density lipoprotein)	LDE- ddMTX reduce the lesion size by 65% and the intima -media ratio by 2-fold. Reduce the intimal macrophage invasion by 67% and apoptotic cell by 88%. Downregulate the pro-inflammatory genes such as (TNF-α, MCP-1, IL-1β, MMP-9, etc.) within the intimal blood vessel and upregulate the anti-inflammatory gene IL-10	(Bulgarelli et al., 2013)
Immune-modifying NPs (IMPs), derived from polystyrene, microdiamonds, and biodegradable PLGA	Monocytes/macrophages	Negatively charged IMPs were taken up by inflammatory cells, directed them to the spleen for apoptosis and no longer travel to sites of inflammation.	(Getts et al., 2014)
Liposomal dexamethasone	Macrophage phagocytosis	Dexamethasone-loaded liposomes inhibited migration of monocytes, precursor cells for tissue macrophages, inhibit proinflammatory activation depends on TNF-α and IL-6.	(Bartneck et al., 2014)
Reconstituted HDL (rHDL) loaded encapsulated with statin or Gd	Macrophages	Statin-rHDL accumulated in macrophages and suppress the inflammatory response through the inhibition mevalonate pathway of in atherosclerotic lesions. Plaque inflammation progression was inhibited by 3- month low dose of statin-rHDL.	(Duivenvoorden et al., 2014)
PLGA-b-PEG NPs encapsulating liver X agonist GW3965 (NP-LXR)	Macrophage phagocytosis	NP-LXR reduced the CD68-positive macrophage content in lesions by (50%) without increasing triglycerides or total cholesterol in the plasma and liver.NP-LXR decrease the inflammation and increase the LXR gene expression compare to free GW3965.	(Zhang et al., 2015)
PLGA-PEG-Col IV NPs incorporated with anti-inflammatory cytokine IL-10	Collagen-IV binding peptide	Col-IV IL-10 NP22 showed more potency than the native IL-10 in reducing acute inflammation in a peritonitis model. Col-IV IL-10 NPs prevented plaque formation, increased cap thickness, and decreased necrotic cores.	(Kamaly, Fredman, et al., 2016)
PEG-PEI miRNA NPs (microRNA (miR-146a and miR-181b) encapsulated within silicon microparticles (ESTA-MSV)	E selectin target peptide	ESTA-MSV-miRNA inhibits chemokine and monocyte adhesion to endothelial cells. ESTA-MSV-miRNA decreased plaque size and macrophage infiltration while increased collagen deposition and SMC migration.	(Ma et al., 2016)
Endothelial cell functionalized with PLA-based magnetic NPs (PLA-MNP)	Aortic endothelial cells	15 minutes of magnetic field allowed the endothelial cells to attach and repopulate the damaged area thereby restore the blood flow	(Adamo et al., 2016)
Gold nanosphere coated with anti-miR‐712 coated and VCAM-1 binding peptide	VCAM-1	Inhibit pro-atherogenic miRNA	(Sun et al., 2016)
PEG-coated SWNTs-loaded with fluorescent probe Cy 5.5 and inhibitor of CD47-SIRPα signaling axis	Macrophages	Pro-efferocytosis nanotherapy reduced plaque burden in atheroprone Apo E**^−/−^** mice by restoring the lesional phagocytosis of apoptotic and non-apoptotic cellular debris	(Flores et al., 2020)
Mesoporous silica NPs with CD9 coated with hyaluronic acid	Macrophages and endothelial cells	Reduce reactive oxygen species level, high density lipoprotein oxidation, and production of TNF-α and IL-6	(Pham et al., 2021)

## 5. Conclusion

The major concern that exists in the treatment strategies for CVD is the prevailing medications currently being prescribed additionally have major side-effects. The best way to combat this issue by utilizing nanocarriers that encapsulate bioactive molecules and nutraceuticals as a futuristic approach to atherosclerotic therapies. The application of nano-based therapies to move forward in clinical trials necessitates having a patient population that might greatly derive therapeutic benefits and evaluates the therapeutic dose margin of the nanoformulation. The application of clinically relevant models for the initial assessment of biomarker analysis and advanced molecular imaging methods will additionally support the actions of the target-driven NDDS to have the success stories in the clinical trial purposes. Moreover, *in vivo* studies on the physicochemical behavior of nanoparticulate systems such as stability during circulation and their relative uptake by certain cell types (intraplaque macrophages) is incumbent to have a better perspective regarding their efficacy. Along with the physiological behavior of the NPs, the growing concerns for the production of NPs in terms of scalability, reproduction, cost-effectiveness, storage stability, and safety are other parameters that affect the overall perspective of the clinical translation of nanomedicine from bench-to-bedside. Since nutraceuticals have an advantage over conventional drugs in terms of safety profile the nano-nutraceutical formulations could be taken as oral supplements over a lifetime to prevent or treat atherosclerosis without posing any deleterious risks to the overall health of the patients. The future directions of nano-nutraceutical formulations also should be to determine the added health benefits of the synergistic effects of different nutraceuticals when encapsulated together into a single nanoformulation. This futuristic strategy would help to harness the potential benefits of different natural compounds to exert their targeted actions on the different stages of the atherosclerosis process. Moreover, a proper treatment regimen should be delineated including dosage, duration, stage of the disease, and patient stratification based on clinical trial evidence and extensive molecular mechanisms of the nutraceutical nanomedicines to predict a better understanding of the treatment outcomes. The continuous trend of developing novel and complex nanosystems, for example, cell-derived biomimetic NPs that has the combined advantage of synthetic NPs and harboring a natural bio-membrane incorporating new target molecules offers hope in the treatment of atherosclerosis. Though this approach of biomimetic NPs comes with challenges like genetic risks, low recovery rates, limited therapeutic dosage, stability, and maintenance of the integrity of the cell membrane future research possibilities aspires to greatly benefit atherosclerotic therapies. Polarizing pro-inflammatory M1 macrophages to anti-inflammatory M2 macrophages, focusing on enhancing the nano delivery of nutraceuticals, and the application of combination therapies with the existing lipid-lowering drugs is light at the end of the tunnel for nanotechnology to revolutionize atherosclerotic treatments.
